# Meetings of Societies

**Published:** 1908-12

**Authors:** 


### MEETINGS OF SOCIETIES.

### Bristol Medico-Chirurgical Society.

*Annual Meeting, October 14th*, 1908.

After a vote of thanks to the retiring President, Dr. Henry Waldo, had been passed, Dr. Michell Clarke took the chair and gave an introductory address (see p. 289). Later a cordial vote of thanks was given to Dr. Clarke for his able and interesting address.

The Honorary Secretary, Dr. J. A. Nixon, read his annual report. The balance in hand was £94 4s. 2d. The Society had lost one member through death, and seven members had resigned. Eleven new members had been elected. Mr. H. F. Mole had resigned the Secretaryship, to the great regret of the members of the Society. The session had been marked by the inauguration of a system by which the circulation of the books in the library among the members was permitted. The constitution of the Committee of the Society had been altered, and would in future consist of four past-presidents, and a greater number of elected members than previously.

The Editorial Secretary of the *Journal*, Mr. James Taylor, read his annual report.

Mr. C. K. Rudge, the Hon. Librarian, read the following report of the Library Committee of the Bristol Medico-Chirurgical Society, for the year ending Sept. 30th, 1908:—

The Library of the Bristol Medico-Chirurgical Society now consists, including duplicates, of 11,002 volumes. 283 volumes have been added during the year (of these 119 volumes were sent to the *Journal* for review), and 196 volumes have been sold. The Library is now in regular receipt of 187 periodicals.

The Bristol Medical Library, to which members of the Society have access, consists of 21,253 volumes, and it is in regular receipt of 244 current periodicals. These are contributed by the constituent libraries in the following manner:

BOOKS.

| | |
|---|---|
| Bristol Medico-Chirurgical Society .. .. | 11,002 |
| Faculty of Medicine, University College | 8,445 |
| Bristol Royal Infirmary .. .. .. .. .. | 1,192 |
| Bristol General Hospital .. .. .. .. .. | 614 |
| Total .. .. | 21,253 |

PERIODICALS.

| | |
|---|---|
| Medico-Chirurgical Society .. .. .. .. .. | 187 |
| Faculty of Medicine .. .. .. .. .. .. | 57 |
| Total .. .. .. | 244 |

The past year marks an eventful epoch in the history of the Library, for during this period several important changes have been effected in the constitution and management, which, it is hoped, will increase its efficiency and usefulness, and also add largely to the Society's roll of members. The Committee, while fully recognising the value and importance of a reference library, and after studying the conditions existing in twenty of the medical libraries in Great Britain which all allow their books to circulate among the members, have arranged that our members may borrow books under certain restrictions, without any increase in their annual subscription.

To meet the requirements of members who wish to borrow the most recent books as soon as they are published, the Society has become a subscriber to Lewis's Library.

The Committee have now under their consideration a scheme for establishing an "Association of British Medical Libraries," one function of which will be to supply the Libraries incorporated in the Association with the most recent periodicals, British and foreign, dealing with the various departments of medical science.

The announcement of the resignation of Mr. James Taylor of the office of Editorial Secretary of the *Journal*, after ten years' service, was heard with much regret. Dr. James Swain was chosen as President-elect, and four members of the Society were elected to serve on the Committee.

The following are the officers for the Session 1908–9: President—J. Michell Clarke, M.A., M.D. Cantab. President-elect—J. Swain, M.D., M.S. Lond. Committee (*Ex officio*)—H. Waldo, M.D. Aberd. (retiring President); R. Shingleton Smith, M.D.Lond. (Editor of *Journal*); J. A. Nixon (Editorial Secretary); C. K. Rudge, M.R.C.S. (Hon. Librarian). (*Elected*)—J. P. Bush, C.M.G. (Ex-President); J. Taylor, M.R.C.S. (Ex-President); B. M. H. Rogers, M.D.Oxon.; P. Watson Williams, M D.Lond.; G. Parker, M.D.Cantab.; I. Walker Hall, M.D.Vict.; F. Lace, F.R.C.S.; J. Lacy Firth, M.D., M.S.Lond. Hon. Secretary—J. A. Nixon, M.B.Cantab.

---

### LAWS OF THE BRISTOL MEDICO-CHIRURGICAL SOCIETY.

1.—The Society shall be called "THE BRISTOL MEDICO-CHIRURGICAL SOCIETY."

2.—The Society shall consist of—

(*a*) A President, President-elect, retiring President, Honorary Secretary, Honorary Librarian, the Editor and the Editorial Secretary of *The Bristol Medico-Chirurgical Journal* who, *ex-officio*, together with eight elected members shall form the Committee. Of the eight elected members two only shall be Past Presidents.

(*b*) Honorary Members not exceeding ten in number.

(*c*) Corresponding Members.

(*d*) Ordinary Members.

3.—The Officers and Committee shall be elected annually, by ballot if demanded, the President and President-elect not being eligible for election to the same office two years in succession.

4.—Any registered Medical Practitioner desirous of entering the Society shall be proposed and seconded by Members at one meeting, and shall be voted for by show of hands at the following meeting, one adverse vote in five to exclude. The name, residence and professional qualifications of any Candidate for Membership, together with the names of the proposer and seconder, shall be announced in the notice convening the meeting at which the election is to take place.

5.—If the conduct of any Member be deemed prejudicial to the welfare of the Society, on notice signed by not less than ten Members being given to the Secretary, the matter may be brought under the consideration of the Society; and if on a show of hands it be so voted by a majority of two-thirds of the Members present, that Member shall be expelled.

6.—A Meeting shall be held on the second Wednesday in each month, from October to May, inclusive, when the chair shall be taken at 8 p.m. If patients are to be shown, the Honorary Secretary shall summon Members for 7.45 p.m., or such earlier hour as he may deem necessary. The meeting in October shall be the *Annual Meeting*, at which the election of Officers and Committee shall take place.

7.—The subscription to the Society shall be One Guinea per annum, payable in advance; this sum shall include the subscription to the *Journal* and the use of the Library, with the privileges appertaining to the circulation of Library books.

8.—Any Member may introduce a Visitor, with the permission of the President.

9.—The following shall be the order of business:—

(*a*) The minutes of the preceding meeting to be read and confirmed.

(*b*) New Members to be voted for.

(*c*) Any candidates for membership to be proposed and seconded.

(*d*) Any miscellaneous business permitted by the President.

(*e*) Remarks upon patients shown.

(*f*) Remarks upon specimens exhibited.

(*g*) Papers to be read and discussed.

10.—The President shall have unlimited authority on all points of order, and in addition to his vote as Member, be entitled to a casting vote.

11.—The Secretary shall give notice of Meeting to all the Members ; shall keep an accurate record of the proceedings of the Society and of the Committee in the books kept for the purpose ; shall receive subscriptions, and pay all debts incurred by the Society ; and shall furnish a report of the proceedings and of the financial position of the Society at each Annual Meeting.

12.—The Committee shall assemble for business as often as any special matter may require it — seven Members constituting a quorum. They shall have the control of all the funds of the Society, and they shall make such regulations as the welfare of the Society may demand.

13.—The Society shall publish a Journal called *The Bristol Medico-Chirurgical Journal*, directed by an Editorial Staff, the Officers and Members of which shall be appointed annually by the Committee of the Society.

14.—The Society shall maintain a Library called "The Library of the Bristol Medico-Chirurgical Society," and shall appoint three of its members, in addition to the President and Honorary Secretary, who shall represent the Society upon the Committee of the Bristol Medical Library, and be responsible for the books and other property appertaining to the Library and Reading Rooms of the Society.

15.—Two Auditors shall be appointed at the Meeting of the Society in May, in order that the accounts may be audited before the Annual Meeting.

16.—The Laws of the Society shall be printed, and a copy sent to each Member. No alteration in any Law shall be made until it has been notified in the circular convening a Meeting, carried at that Meeting, and confirmed at the succeeding Meeting.

H. Waldo, *President*.
J. A. Nixon, *Secretary*.

---

### BRISTOL MEDICAL LIBRARY.

---

### By-Laws for Borrowing Books.

(*a*) Every Member shall be entitled to borrow books from the Library, and may have three volumes or periodicals in his possession at one time, one of which may be from Lewis's Library.

(*b*) The Member shall give a receipt to the Librarian for each volume so borrowed, and shall be responsible for any damage done to it while in his possession, and shall replace it if lost ; he shall also discharge the cost of carriage on any books sent out by carrier or post.

(*c*) A Member shall not retain a volume for a longer period than 14 days; for detention beyond this period a fine of 3d. per day will be charged on each volume, and he shall not be entitled to borrow another book until the fine is paid.

(*d*) Current periodicals may only be taken out from Saturday noon till 10 a.m. on the following Monday.

(*e*) The following books are for reference only:—

Old and rare volumes.
Dictionaries (language and medical).
Encyclopædias.
Allbutt's *System of Medicine,* and similar systems.
Any student's text-book, which is not a duplicate.
Large atlases and plates.

C. King Rudge,
*Hon. Librarian.*

---

### RECEIPT AND EXPENDITURE ACCOUNT OF THE BRISTOL MEDICO-CHIRURGICAL SOCIETY, SESSION 1907-8.

| | £ | s. | d. |
|---|---|---|---|
| Carried forward from 1906–7 | 120 | 18 | 7 |
| Members' Subscriptions, 1907–8 | 136 | 10 | 0 |
| Interest on Deposit Notes | 3 | 15 | 7 |
| | £261 | 4 | 2 |

| 1907. | £ | s. | d. |
|---|---|---|---|
| Oct. 13.—Stamps | 1 | 0 | 0 |
| Nov. 11.—Mr. Farr, Grant | 5 | 5 | 0 |
| ,, 11.—Stamps | 1 | 0 | 0 |
| 1908. | | | |
| Feb. 24.—Stamps | 1 | 5 | 0 |
| Sept. 2.—Stamps | 1 | 5 | 0 |
| Sept. 12.—Grant to Library | 50 | 0 | 0 |
| ,, 26.—To *Journal* | 41 | 0 | 0 |
| ,, 28.—Sundries | 3 | 7 | 9 |
| ,, 28.—For use of rooms, telephone, &c. | 57 | 6 | 0 |
| ,, 29.—Printing | 5 | 1 | 0 |
| Balance | 94 | 14 | 5 |
| | £261 | 4 | 2 |

---

*November 11th.*

Dr. Michell Clarke, President, in the Chair.

Dr. E. C. Williams showed a child, aged 2 years, who had recently recovered from **Basic Meningitis with temporary Amaurosis.** During the course of the disease there had been complete loss of sensibility to light without any visible change in the ophthalmoscopic appearances. The case had markedly benefited from small doses of liquor morphinæ, administered regularly.

Recovery had left the child unable to walk or talk, but the vision was apparently good. He regarded the case as an example of that form of posterior basic meningitis during the course of which the child appears to lose its sight without any discoverable ophthalmoscopic change. The children at a later date often regain both their sight and health. They are usually below two years of age.—Mr. MOLE, in discussing the case, suggested that possibly the child was deaf as the result of the meningitis, and hence was unable to regain the lost art of speech.—Dr. BERTRAM ROGERS said that his experience did not agree with that of Hutchison (previously cited), that about half the cases of posterior basic meningitis make a more or less complete recovery.—Mr. CYRIL WALKER suggested that the amaurosis was due possibly to a retro-ocular neuritis; the loss of pupil reflex to light pointed to this conclusion.—Dr. EDGEWORTH referred to the treatment of this disease by serum, which had reduced the mortality in the Belfast epidemic 30 per cent., questioning whether the same serum would prove efficacious in the sporadic cases. The meningococcus in the two varieties, although morphologically and culturally identical, gave different agglutination and opsonic reactions, so that it was probable that the serum of the epidemic variety would fail with the sporadic. He had seen in three or four cases very good results from the morphia treatment advocated by Barr.—Prof. WALKER HALL thought that, admirable as Flexner's serum had proved in some epidemics, the correct plan to follow was that of preparing a serum from individual cases or epidemics wherever practicable.

Dr. WATSON WILLIAMS showed a patient upon whom he had operated for **Suppuration in the Frontal Sinus** by his osteoplastic flap method. The result had been excellent, not only from the point of view of relieving the symptoms, but also as regards the cosmetic effect, the scar being barely visible.—Mr. LACY FIRTH agreed that the operation was a very good one; he had found that it gave excellent access to the ethmoidal region, and left no disfigurement, except an almost invisible scar.

Mr. JAMES TAYLOR read a paper on **Some Fallacies in Skiagrams,** which he illustrated by actual mistakes, showing how a fracture might escape notice completely if photographed in a single plane. Tuberculous masses in the kidney simulating calculi and other examples were shown.—Dr. JAMES SWAIN insisted on the importance of such facts being published from the medico-legal aspect: he said that frequently the result of treatment of a fracture looked bad in a skiagram, while clinically no inconvenience was experienced.—Dr. WATSON WILLIAMS emphasised the value of stereoscopic skiagrams, which cleared up many difficulties.—Dr. BOWKER spoke of the misleading appearance of exostoses in some cases of suspected injury.—

Mr. Hey Groves, referring to the transparency of callus, said that the patients might, by seeing a skiagram, be led to suppose that there was no union between the ends of a fractured bone.—Mr. A. L. Flemming said that during the South African War he had come to realise the value of stereoscopic skiagrams.

Dr. H. L. Ormerod read notes of a case of a **Double Monster.** A full-time birth in a *primipara*, aged 25; the two embryos were joined by a broad isthmus from manubrium sterni to umbilicus (thoracopagus); their total weight was 11¾ lbs., sex female. When he arrived the mother had been in labour eighteen hours, the waters had broken one hour previously, the pains were vigorous, and the abdomen was unduly prominent. The cervix was well dilated, and the presentation was a third vertex: progress had come to a standstill. The head was delivered with forceps, but the body would not follow; vaginal examination revealed some abnormal prominence over the chest. Without other assistance than that of the midwife, he succeeded in bringing down the arms, and subsequently the legs and breech: he then found there was a second foetus attached. He brought down the legs of the second child, and had little difficulty in delivering the body and after-coming head. The monster died during delivery. The mother made an uninterrupted recovery, the perineum even having escaped laceration.

Dr. T. M. Carter read notes of a case of **Rupture of Cerebral Aneurism in a girl aged 16** (*vide* p. 322). The early symptoms simulated hysteria, death occurred in a few hours preceded by absolute coma. The autopsy revealed an extensive hemorrhage at the base of the brain.—Mr. Munro Smith said that he had seen the patient in the earlier stages of her fatal seizure, and had been misled into regarding the symptoms as hysterical.—Dr. Charles had seen her after she had become comatose, and was led to diagnose cerebral hemorrhage from the ophthalmoscopic appearances, which revealed a very early stage of optic neuritis. He mentioned another case which he had seen where a similar condition was responsible for sudden death.—Professor Walker Hall described the *post-mortem* findings, and showed direct colour photographs of microscopic sections from the dilated vessel which had burst. He was unable to say whether the vessel was an artery or vein. Apart from this lesion, there was general hypoplasia of the cardio-vascular system, and an abnormal fluidity of the blood which had only clotted round the ruptured vessel, but in no other part of the body.—Dr. Edgeworth said that whilst pathologist at the Royal Infirmary he had met with several cases of basal hemorrhage in young adults, in which, after careful examination, the source of hemorrhage could not be found.—Dr. Alexander inquired whether there was any reason for supposing that Arthur Hallam's untimely death from a "determination of blood to the brain" was an instance of this condition.—The President remarked that the mistake of at first confounding such a case with hysteria would undoubtedly be made again. He asked whether there was any communication between the auricles or ventricles associated with the cardiac hypoplasia ; also whether there was any *ante-mortem* clot which could have been dislodged and given rise to aneurism in the brain. The answer was in the negative to both these questions.

Eighteen candidates were proposed and elected members of the Society.

J Lacy Firth.
J. A. Nixon, *Hon. Sec.*

